# Cationic Interstitials: An Overlooked Ionic Defect in Memristors

**DOI:** 10.3389/fchem.2022.944029

**Published:** 2022-07-08

**Authors:** Zhemi Xu, Peiyuan Guan, Tianhao Ji, Yihong Hu, Zhiwei Li, Wenqing Wang, Nuo Xu

**Affiliations:** ^1^ College of Chemistry and Material Engineering, Beijing Technology and Business University, Beijing, China; ^2^ School of Materials Science and Engineering, University of New South Wales, Sydney, NSW, Australia; ^3^ College of Computer, National University of Defense Technology, Changsha, China; ^4^ College of Electronic Science and Technology, National University of Defense Technology, Changsha, China

**Keywords:** resistive switching (RS), cationic interstitials, metal oxides, memristor, conductive filament

## Abstract

Metal oxide-based memristors are promising candidates for breaking through the limitations in data storage density and transmission efficiency in traditional von Neumann systems, owing to their great potential in multi-state data storage and achievement of the in-memory neuromorphic computing paradigm. Currently, the resistive switching behavior of those is mainly ascribed to the formation and rupture of conductive filaments or paths formed by the migration of cations from electrodes or oxygen vacancies in oxides. However, due to the relatively low stability and endurance of the cations from electrodes, and the high mobility and weak immunity of oxygen vacancies, intermediate resistance states can be hardly retained for multilevel or synaptic resistive switching. Herein, we reviewed the memristors based on cationic interstitials which have been overlooked in achieving digital or analog resistive switching processes. Both theoretical calculations and experimental works have been surveyed, which may provide reference and inspiration for the rational design of multifunctional memristors, and will promote the increments in the memristor fabrications.

## Introduction

As the memories and CPUs are separated in the current von Neumann computer system, the data have to be transferred between them through the limited bandwidth buses, which limits the time and energy efficiencies in the data processing. Such issue could be addressed by achieving an in-memory computing paradigm, for which the memristor is a suitable device because of its higher data storage density (Cheng et al., 2017; Chen et al., 2019) and excellent physical characteristics of conditional switching and physical MAC operation (Yang et al., 2017; Zhou et al., 2019; Xu et al., 2021; Li et al., 2022). Meanwhile, they can also bridge various electrical devices and be applied in energy storage, remote sensing, low-power applications, *etc*. (Chen, 2017; Han et al., 2020; Wang et al., 2020; Zhang and Sun, 2021) Thus, memristors are crucial for non-volatile memory, logic operations, Internet of Things, and neuromorphic computing in the big data era (Qin et al., 2020; Xu et al., 2020; Yao et al., 2020; Huang et al., 2021).

The memristor is a two-terminal electrical device that regulates the flow of electrical current in a circuit and remembers the amount of charge that has previously flowed through it even after removing the bias voltage (Fu et al., 2020). The original concept for memristors was proposed by Leon Chua in 1971, which was described as a nonlinear, passive two-terminal electrical component that linked electric charge and magnetic flux (Chua, 1971). This conceptual device has been firstly linked to a kind of physical resistive switching device (ReRAM) by HP labs in 2008 (Strukov et al., 2008). Nowadays, the definition of memristor has been broadened to the arbitrary form of non-volatile memory with the foundation principle of resistance switching.

So far, the state transition phenomenon in different material systems is employed to trigger the resistive switching (RS) behavior and further construct the different types of memristors. Except for the well-studied RS behavior in metal-oxide materials (Illarionov et al., 2020; Liu et al., 2021; Wang et al., 2021), phase change materials (Hazra et al., 2021), organic materials (Chen, 2017; Cheng et al., 2017), ferroelectric materials (Guan et al., 2017), and magnetic materials (Park et al., 2018) are also reported to exhibit the macroscopic RS behavior because of the transition of crystalline phase, ferroelectric polarization, and spin polarization respectively. Among these numerous material systems, the metal-oxide-based memristors are promising owing to their low cost, simple process, and high compatibility with complementary metal-oxide-semiconductor (CMOS) technology (Mohammad et al., 2016; Illarionov et al., 2020; Nili et al., 2020).

However, compared to the basic metal-oxide-semiconductor (MOS) transistor which is the foundation device of constructing the current computing system, the memristor is still suffering from the relatively low reliability caused by device fluctuation, limited stability, and durability to maintain the resistance value or to improve repeated erasable times. Therefore, rational design and optimization of the memristor active layer through material engineering for enhancing the performance of memristors are expected. The works on metal-oxide-based RS have been mainly focused on the migration or ionization of oxygen vacancies (V_O_) or cations from active electrodes (Waser et al., 2009; Chen et al., 2013; Kamiya et al., 2014; Tang et al., 2015). However, due to the high mobility of V_O_ and relative low endurance of cation-based resistive random-access memories (RRAM), it is a challenge to maintain stable intermediate states, which greatly limited the applications of memristors in multi-level RS and artificial synapses.

Compared to Vo and cations from electrodes, another common ionic defect in metal oxides, cationic interstitial (C_int_) can also contribute to the successful RS, which has been less focused on previously. In the limited reports, C_int_s have shown great potential in enhancing the performance of memristors with better stability and endurance, higher ON/OFF ratio, lower operation voltage, *etc*. Multi-level RS and synaptic RS have also been realized *via* modulating C_int_s in memristors. Hence, in this mini-review, we focused on studying the C_int_-induced RS behaviors from the previous reports. Both theoretical and experimental works have been investigated, which may provide reference and inspiration for the rational design of multifunctional memristors from a new perspective and may shed some light on the increments in memristors.

## Review of the Works Related to the First-Principle Studies

First-principles calculations have been always employed to investigate the mechanism of the RS behavior through some calculations in terms of formation energy, the density of states, partial charge densities, *etc*. For C_int_s-induced RS, there are two main contributions from C_int_s to realize or enhance the RS: forming a conductive path and promoting charge transfer in the metal oxides. Related works have been summarized in Table 1.

**TABLE 1:** Theoretical works on the C_int_-induced RS behavior.

Materials	Interstitials	Effects	Ref
Ta_2_O_5_	Cu_int_	Forming conductive path	Gu et al. (2010)
TiO_2_	Ti_int_ or Zr_int_	Promoting charge transfer	Li et al. (2015)
CeO_2_	Ti_int_ or Zr_int_	Forming conductive path	Hussain et al. (2018)
Ta_2_O_5_	Ta_int_	Forming conductive path	Zhu et al. (2016)
HfO_2_	Au_int_ + Vo	Forming filaments	Tan et al. (2018)
TiO_2_	Ti_int_ + Vo	Forming net dipole moment	Abdelouahed and Mckenna, (2015)

Gu et al. have compared the formation possibility of conductive paths with Cu interstitials (Cu_int_) and Vo in the Ta_2_O_5_ atomic switch through first-principles studies (Gu et al., 2010): Figure 1A shows that Cu_int_ can form an effective conduction channel in the Ta_2_O_5_, as the formation of Cu_int,_ may connect the two adjacent Ta-O planes through the simulation process, while Vo failed to form such a conductive channel. But it should be noted that the conductive path formed by C_int_s is sensitive to the concentrations of interstitials, which need to be tuned carefully in the experiments.

**FIGURE 1:**
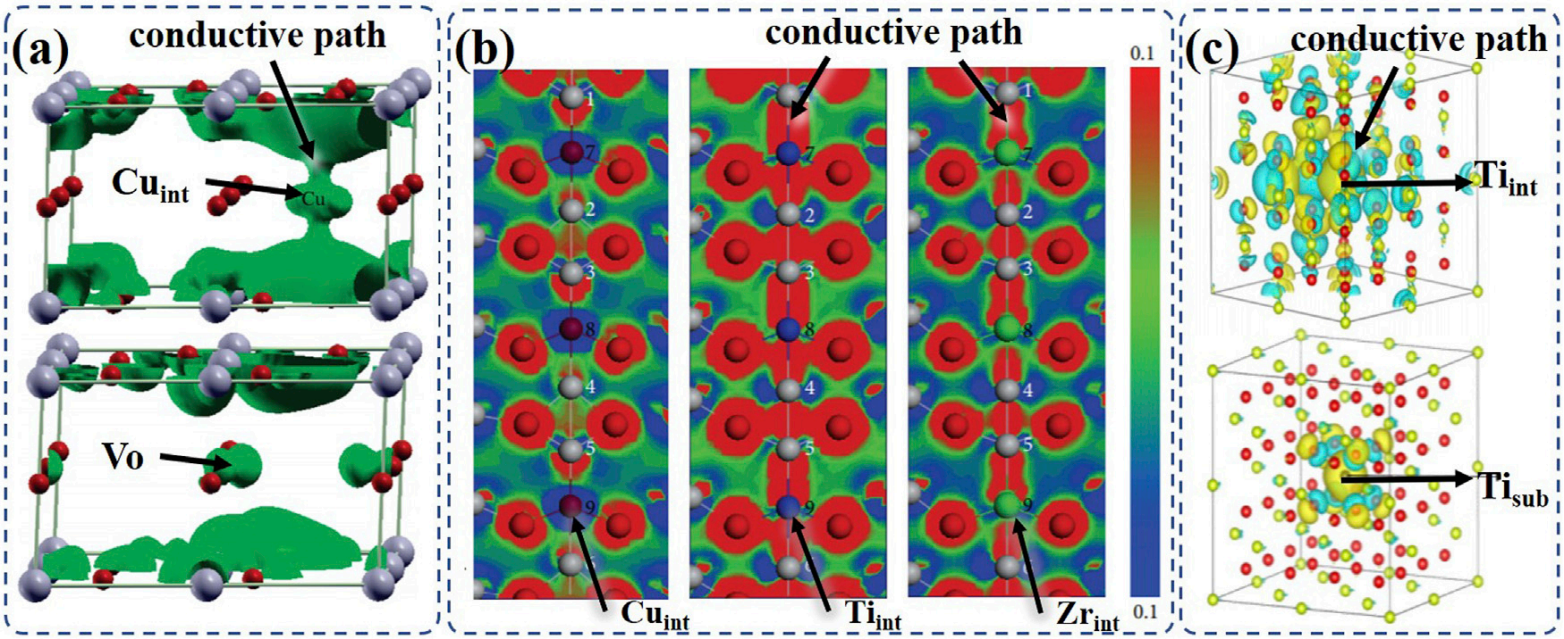
**(A)** Isosurface plot of the partial charge density corresponding to the defect state induced by the interstitial Cu_int_ and Vo in Ta_2_O_5_ (reproduced with permission (Gu et al., 2010). Copyright 2010, American Chemical Society); **(B)** deformation electron density in [110] for the defected TiO_2_ with the Cu_int_, Ti_int,_ and Zr_int_ (reproduced with permission (Li et al., 2015). Copyright 2015, Lei Li et al.); **(C)** isosurface plots of Ti_int_ and Ti_sub_ (reproduced with permission (Hussain et al., 2018). Copyright 2018, Springer-Verlag GmbH Germany).

The formation of the conductive path with C_int_s is sensitive to the valences of the doped cations as well. Li et al. have systematically calculated the TiO_2_ and ZrO_2_ with different C_int_s and investigated how C_int_s with different valence states may affect the transport coefficients (Li et al., 2015). Figure 1B illustrates the deformation electron densities for the TiO_2_ with Cu_int_, Ti_int_, and Zr_int_, respectively. The blue region around Cu_int_ indicates that the loss of e^−^ from Cu could form ionic bonds with nearby O atoms, while such a phenomenon has not been observed in the situation of Ti_int_ and Zr_int_. The calculation results indicate that the transport coefficients of the materials with Ti_int_ and Zr_int_ are higher than that with Cu_int_. To optimize the RS behavior, the doping of metals with +4 or higher valences could be employed as it may enhance the transport properties.

Similarly, Zr_int_ and Ti_int_ in CeO_2_ and Ta_int_ in Ta_2_O_5_ have also been confirmed to contribute to the formation of conductive paths in memristor. As it is shown in Figure 1C, Ti_int_ can form a more obvious conductive path in CeO_2_ compared to the Ti substitution (Ti_sub_). And then, Ti_int_ and Zr_int_ have been introduced in experiments and successfully improved the RS performance of CeO_2_ RRAMs (Hussain et al., 2018). Zhu et al. compared the V_O_ and Ta_int_ in the Ta_2_O_5_-based RRAM and confirmed the contribution of Ta_int_ in realizing RS under oxygen-poor conditions (Zhu et al., 2016). Thus, it is concluded that the C_int_s can introduce more defect states to above metal oxides than that of Vo, and the C_int_-induced RS can be enhanced under an electric field.

The synergistic effects of Vo and C_int_s for achieving RS have also been identified in memristors. In the Au-doped HfO_2_, it has been confirmed that both Vo and Au_int_ are involved in the formation of conductive filaments (Tan et al., 2018). Similarly, Abdelouahed et al. compared the TiO_2_ with Vo and Ti_int_ and revealed the co-formation of both defects, which induced a net dipole moment, and enhanced RS behavior under an electric field (Abdelouahed and Mckenna, 2015).

## Review of Experimental Works

C_int_-induced or enhanced RS behavior in memristors has also been confirmed in experimental works (as summarized in Table 2).

·The formation of C_int_s and C_int_-induced RS behavior

**TABLE 2:** Experimental works on the C_int_-induced RS behavior.

Materials	Interstitials	C_int_ forming conditions	Effects	Ref
Cu_x_O	Cu_int_	Annealing in Ar environment	Enhanced RS	Rehman et al. (2018)
ZnO_x_	Zn_int_	Sputtering under high oxygen partial pressure	Change bipolar (with O_int_) into unipolar (with Zn_int_) RS	Wu et al. (2014)
ZnO/Al_2_O_3_	Zn_int_	PLD, rapid thermal annealing	Change TCSC conduction (with Vo) into diode-like RS (with Zn_int_)	Sekhar et al. (2015)
NiO:SnO_2_	Ru_int_ + Al_int_	Sol-gel, Ru, and Al co-doping	Enhanced RS with a higher ON/OFF ratio	Li et al. (2014)
CeO_2_	Ti_int_	Depositing Ti as a buffer layer in CeO_2_/Ti/CeO_2_	Improved stability, endurance, and ON/OFF ratio, lowered SET voltage	Rana et al. (2017)
SnO_2_	Mn_int_	Hydrothermally synthesized Mn-doped SnO_2_	Intrinsic multi-level RS, improved stability and endurance	Xu et al. (2018)
TiO_2_	Ti_int_ + Vo	Thermally-induced self-doping and phase transformation	Improved stability, endurance, and ON/OFF ratio, lowered SET voltage	Hazra et al. (2021)
MoO_3_	Mo_int_ + Vo	Hydrothermally synthesized hexagonal MoO_3_	Multi-level RS	Patil et al. (2021)
ZnO	Zn_int_ + Vo	2 wt% Cu-doped ZnO	Enhanced electric controlled RS and light-modulable RS	Saini et al. (2021)
TiO_2_	Ti_int_ + Ag^+^ + K^+^	Fabricate Ag/TiO_2_-LPE/FTO device	Enhanced stability and endurance, lowered SET voltage, bipolar RS	Abbasi et al. (2020)
LaAlO_3_	B_int_	B-doped LaAlO_3_	Enhanced RS behavior, realized ferromagnetic ionic-electronic conductor	Park et al. (2018)

**FIGURE 2:**
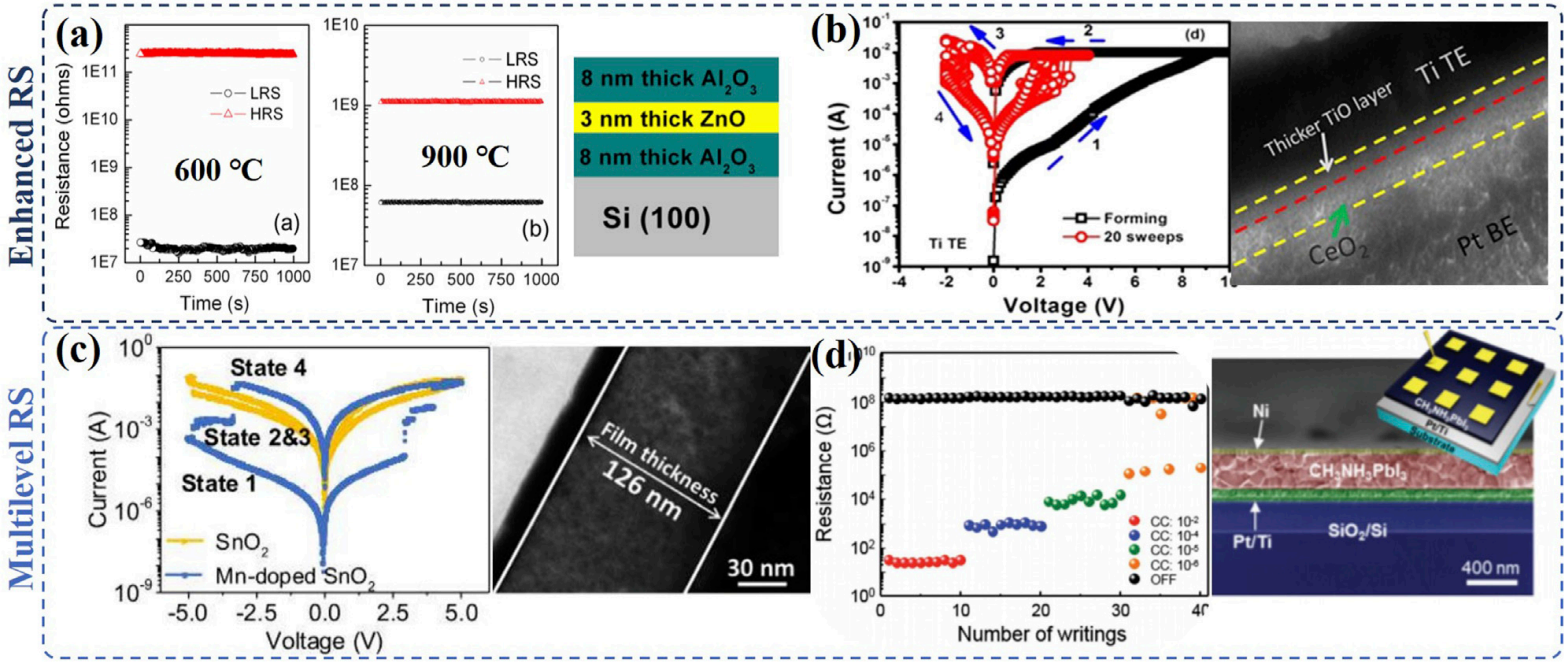
Enhanced RS performance and the cross-section images of the device based on **(A)** Al_2_O_3_/ZnO/Al_2_O_3_ memristors (reproduced with permission (Sekhar et al., 2015). Copyright 2015, Elsevier B.V.) and **(B)** CeO_2_/Ti/CeO_2_ (reproduced with permission (Rana et al., 2017). Copyright 2017, Anwar Manzoor Rana et al.); the multilevel RS and the cross-section images of the device based on **(C)** Mn-doped SnO_2_ (reproduced with permission (Xu et al., 2018). Copyright 2018, Elsevier Ltd.) and **(D)** CH_3_NH_3_PbI_3_ thin films (reproduced with permission (Choi et al., 2016). Copyright 2016, John Wiley & Sons, Inc.).

The C_int_s can be introduced to metal oxides by modifying the synthesis parameters, such as the annealing conditions, oxygen partial pressure, doping concentration, *etc*. For example, by changing the annealing temperature and improving the oxygen concentration during the annealing process, Cu_int_s have been successfully formed in the Cu_x_O, and the RS can be enhanced by tuning the Cu_int_s in the memristors (Rehman et al., 2018). In the sputtering process, by adjusting the oxygen partial pressure, the formation of O_int_ or Zn_int_ could be controlled, and interestingly, it is found that the bipolar and unipolar RS behavior can be tuned by forming O_int_ and Zn_int_ in the Al/ZnO_x_/Al memory device, respectively (Wu et al., 2014). In the pulsed laser deposition (PLD), rapid thermal annealing may also change the defects in ZnO/Al_2_O_3_ memristor: the trap-controlled-space-charge (TCSC) limited conduction mechanism has been observed when Vo dominates, while, diode-like RS behavior has been identified in the case of Zn_int_ dominating. In the latter, the stability, endurance, and ON/OFF ratio of the memristor have been significantly improved as it is shown in Figure 2A (Sekhar et al., 2015). Such transition is ascribed to the formation of ZnAL_2_O_4_ as an interlayer, which acts as the e^−^ trapping/detrapping area and achieved successful RS.

In the memristors fabricated from solution-processed, C_int_s are usually introduced *via* doping. In the NiO:SnO_2_ memristor, Ru_int,_ and Al_int_ can be achieved through Ru and Al co-doping in the sol-gel process (Li et al., 2014). Compared to the Vo induced RS, the RS behavior conducted by Ru_int_ and Al_int_ could be greatly improved and show a larger ON/OFF ratio. The enhanced RS is ascribed to the increased trapped states between the equilibrium Fermi level and conduction band by Ru_int_ and Al_int_ (Li et al., 2014). Mn_int_s have been achieved in SnO_2_ by increasing the Mn-doping level to 12.5 mol% in the liquid-liquid interface hydrothermal process and compared to the pure SnO_2_ with Vo, Mn-doped SnO_2_ memristor shows more effective and stable RS with significantly larger ON/OFF ratio and better intermediate state retention (Xu et al., 2018).

Adding a buffer layer is another method to introduce the C_int_s. As it is illustrated in Figure 2B, Ti_int_s have been introduced by a Ti buffer layer in the TaN/CeO_2_/Ti/CeO_2_/Pt memory device, in which Ti_int_s assisted the formation of conductive filaments in CeO_2_. Compared to the device without the Ti buffer layer, the device’s stability and endurance could be significantly improved alongside the lower SET voltage and larger memory window (Rana et al., 2017). Similarly, by alternately depositing the SnO_2_ layer with Vo and Mn-doped SnO_2_ layer with Mn_int_, Mn_int_s have been introduced to the SnO_2_-based RRAMs, which significantly enhance the RS behavior with a higher ON/OFF ratio and better stability and endurance (Xu et al., 2017).

·C_int_-induced Multi-level RS

Multilevel RS has also been investigated in C_int_-induced memristors. Intrinsic multi-state RS behavior with good endurance and stability has been observed in Mn-doped SnO_2_-based memristor by increasing the Mn-doping concentration, as it is illustrated in Figure 2C (Xu et al., 2018). By comparing the RS behavior of Mn-doping, Al-doping, and In-doping in SnO_2_ together with the XPS results and the calculated defect formation energies, the multi-level RS has been ascribed to Mn_int_ instead of Vo. Iodine interstitials induced multi-level RS has also been achieved in the Ag/CH_3_NH_3_PbI_3_/Pt cells as shown in Figure 2D (Choi et al., 2016). Owing to the relatively low activation energies, the migration of I_int_ enables filament formation and annihilation at a relative operation voltage.

·Synergistic RS induced by C_int_ and other defects

Furthermore, the synergistic effect of C_int_s with other ionic defects in memristor has been confirmed in experiments more than the theoretical results above (Lee et al., 2021). The synergistic effect of Ti_int_ with Vo has been confirmed in the Au/TiO_2_ nanotube/Ti memory (Hazra et al., 2021). In the Ti/MoO_3_/FTO memory cell, it is identified that Mo_int_, surface defects, and Vo have contributed together to the multilevel RS behavior (Patil et al., 2021). Zn_int_ together with Vo enables the formation and rupture of conducting filaments in the Cu-doped ZnO, and both electric controlled and white light modulated RS has been achieved (Saini et al., 2021). Similarly, Vo and I_int_ assisted RS *via* a Schottky barrier tuning has also been verified in the Au/CH_3_NH_3_PbI_3_/TiO_2_/FTO memory device (Lee et al., 2021).

**FIGURE 3:**
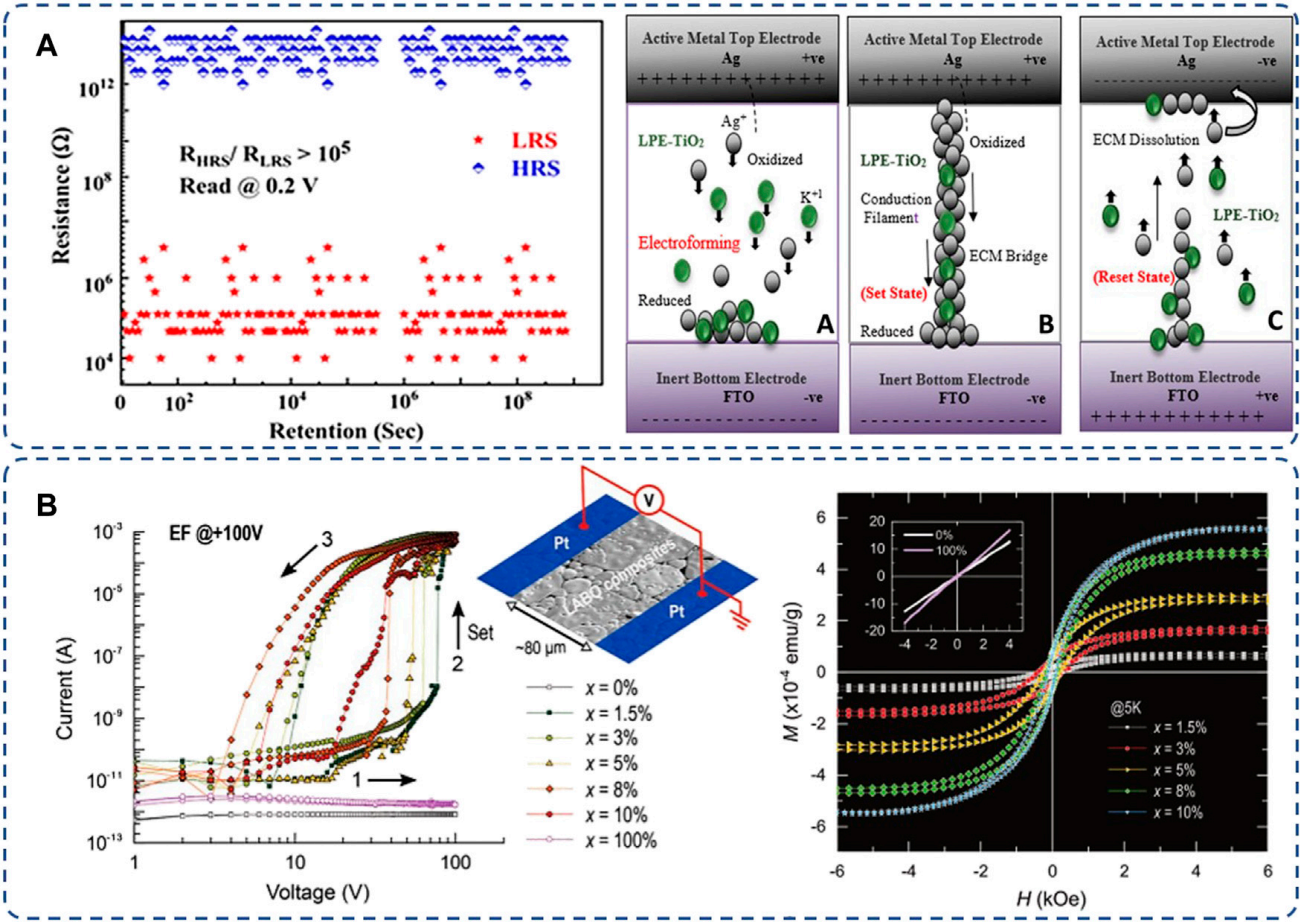
**(A)** Enhanced retention and the schematic of Ti_int_ assisted the conductive filament with Ag^+^ and K^+^ (reproduced with permission (Abbasi et al., 2020). Copyright 2020, American Chemical Society); **(B)** the remarkably enhanced RS and ferromagnetic behavior by B_int_ in LaAlO_3_(1-x):LaBO_3_(x) (reproduced with permission (Park et al., 2018). Copyright 2018, John Wiley & Sons, Inc.).

In addition, C_int_ can act as assistance or a game-changer in metal oxides. Figure 3A illustrates the defect-abundant memory device of Ag/TiO_2_-LPE (known as lime peel extract)/FTO, in which Ti_int_ from TiO_2_, Ag^+^ oxidized from the Ag electrode, and K^+^ from the LPE synergistically contribute to the RS behavior. Ti_int_s provide active paths for cation migrations, which enhanced the stability and endurance of bipolar RS of the memory cell with low operation voltage and high ON/OFF ratio (Abbasi et al., 2020). In the B-doped LaAlO_3_, B_int_s realized charge injection into the neighboring cations, which enables remarkable electrical RS and transformed the oxide into a ferromagnetic ionic-electronic conductor at the same time, as it is shown in Figure 3B. This extends applications of C_int_s to contribute to energy-efficient and spin-based devices (Park et al., 2018).

## Summary and Outlook

In summary, the cationic interstitials induced RS behavior in metal-oxide-based memories has been summarized. For a defect that has been less focused, there are very few reports on the formation, contribution, and mechanism of C_int_-induced RS behavior compared to those on Vo or active electrodes. However, from both theoretical and experimental aspects, the C_int_-induced or enhanced RS behavior has been confirmed in recent years. As discussed above, diversified C_int_s provide more opportunities to tailor the metal oxides for different electronic devices. The rational fabrication of memristors with C_int_s may give rise to remarkable enhancement in RS performance with better stability and endurance, lower operation voltage, higher ON/OFF ratio, faster device speed, *etc*. However, C_int_-based memristors are sensitive to the concentration and valence state of C_int_, which makes the formation of C_int_ in metal oxide synthesis need to be carefully modulated. By adjusting the C_int_s, suitable electric structures would be established in the metal oxides, which helps improve the performance of the electronic devices.
